# Do We Need a Specific Guideline for Assessment and Improvement of Acromegaly Patients Adherence?

**DOI:** 10.3389/fpubh.2021.693409

**Published:** 2021-07-14

**Authors:** Maria Kamusheva, Alexina Parvanova, Yanitsa Rusenova, Silvia Vandeva, Atanaska Elenkova

**Affiliations:** ^1^Department of Organization and Economics of Pharmacy, Faculty of Pharmacy, Medical University – Sofia, Sofia, Bulgaria; ^2^Department of Endocrinology, University Specialized Hospital for Active Treatment of Endocrinology (USHATE) “Acad. Ivan Penchev,” Medical University – Sofia, Sofia, Bulgaria

**Keywords:** acromegaly, adherence, Bulgaria, guideline, health policy

## Abstract

**Background:** Adherence to therapy is one of the most important elements during the therapeutic process ensuring the predefined therapeutic outcomes. The aim is to analyze the need and importance of treatment adherence guideline for acromegaly patients and the possibilities for its development and implementation in Bulgaria.

**Methods:** A set of methods was applied: (1) a literature review in the electronic database for identification of articles and guidelines related to adherence and acromegaly; (2) analysis of Bulgarian legislative documents; (3) a pilot study for assessment of the level of treatment adherence among hospitalized Bulgarian acromegaly patients in 2018; (4) a plan for development and implementation of specific guideline was created entitled BULMEDACRO - BULgarian guideline for MEdication aDherence assessment and improvement in ACROmegaly.

**Results:** No specific guidelines for evaluation, monitoring, reporting and/or improving adherence in acromegaly patients has been found in the literature. Requirements for regular assessment of the level of adherence, application of appropriate methods for improvement and monitoring are not sufficiently formulated and mandatory. The pilot study confirmed that therapy adherence among Bulgarian patients with acromegaly is relatively high as almost 90% of patients report that they strictly comply with their prescribed treatment regimen. It is necessary, however, a specific guideline focused on the methods for assessment and improvement of adherence, in order to ensure monitoring and follow-up of acromegaly patients.

**Conclusions:** Patients with acromegaly should be the focus of specially designed national programs, initiatives and/or guidelines for regular evaluation and improvement of the adherence level. Despite the difficulties and the lack of an adequate legal basis, successive steps initiated by different stakeholder are needed.

## Introduction

Timely and long-term use of prescribed therapy as recommended by the medical specialists is the key to effective control, especially in chronic diseases. A number of studies show that medicines are often not used as prescribed, leading to poor clinical outcomes and higher health care costs. According to published data, ~50% of patients do not take their medications as per their physicians' instructions (1). In certain potentially asymptomatic diseases, such as hypertension, the incidence of non-adherence might reach 80% (2). Addressing the problems with non-adherence to therapy can improve the quality of health care, support better control of chronic diseases, improve therapeutic outcomes and generally reduce the social and economic burden of disease. Non-adherence is one of the most significant challenges facing healthcare professionals, healthcare decision-makers and researchers. Moreover, a recent study pointed out the need for a specific instruments to assure the medication adherence among patients with non-communicable and other chronic diseases during COVID-19 outbreaks (3).

Critical predictors of adherence are trust, understanding, and effective patient - medical specialist relationship. Adherence is the extent to which the patient's behavior matches agreed recommendations from the prescriber (4). Therefore, achieving optimal behavior is a joint process of communication and understanding between the participants in the therapeutic process. Healthcare professionals can improve their patients' behavior when taking medication on an individual and systemic level using variety of methods and approaches and identifying the factors influencing the level of adherence. Following a specific guideline or algorithm can enhance the level of adherence and prevent the consequences of non-adherence. Patients should be actively involved in decisions related to their therapy and be fully consented and informed of the therapeutic process and procedures (5). Being between treatment and outcomes adherence is a crucial element and factor for achieving the desired therapeutic results (1). As it was stated in a Cochrane review “Interventions for enhancing medication adherence” improving therapy adherence might lead to a greater impact on the outcomes than an improvement in treatments (6).

Acromegaly is a rare endocrine disease affecting 2–11 people per million annually and characterized by oversecretion of growth hormone from benign adenoma of the pituitary gland in more than 95% of all cases (7). Early diagnosis, proper treatment, adherence to prescribed therapy and regular monitoring increase the chances of therapeutic success and reduce the risk of disability. According to an epidemiological study (2010), the estimated prevalence in Bulgaria is around 49 cases/million as the annual number of health insured patients with acromegaly or pituitary gigantism is around 200 (8). According to studies, the most common causes of long-term active disease are the patient's refusal to escalate the therapeutic strategy and non-compliance with prescriptions. In a previous study, we identified that the number of studies assessing the level of adherence, consent and persistence to the therapeutic regimen among patients with acromegaly is limited (9). Moreover, there is a lack of systematically conducted real-life studies assessing the level of adherence among acromegaly patients (10). Considering the lack of awareness, training among healthcare professionals and limited resources for adapting suitable practices for improvement and regular assessment of medication adherence especially during pandemic, relevant and urgent activities in this direction are needed (3, 11). Therefore, our aim is to analyze the need of treatment adherence guideline focused on acromegaly patients, give initial pilot results for the level of treatment adherence among Bulgarian acromegaly patients and present initial statements for further development of such guideline for the Bulgarian healthcare system.

## Methods

A literature review limited to English- and Bulgarian-language guidelines and articles published in PubMed and the electronic database of Central Medicine Library, Medical University of Sofia, Bulgaria between January 2000 and January 2021 using the following key words: acromegaly AND adherence AND guidelines.

Several documents adopted in Bulgaria were analyzed for requirements related to adherence assessment and tools for adherence improvement: Good Pharmacy Practice (2020) (12), Good Healthcare Professionals Practice (2020) (13), Good Medical Practice (2013) (14), National Health Strategy 2020 (15), project of National Health Strategy 2021–2030 (16), Pharmacotherapeutic guideline for treatment of endocrinology diseases approved by National Council on Pricing and reimbursement of medicinal products, annex of Regulation No 16 21.11.2019, Ordinance on the terms, rules and procedure for regulation and registration of prices for medicinal products, 2013 (17), Law on the medicinal products in human medicine, 2007 (18) and National Health Insurance Fund requirements for treatment of acromegaly in ambulatory settings (19).

Based on the current adherence policy, education and practice in Bulgaria and the results from the literature search, a plan for development and implementation of specific guideline was created entitled BULMEDACRO - BULgarian guideline for MEdication aDherence assessment and improvement in ACROmegaly. The content of the guideline was developed on the basis of similar guides already published and implemented in the practice.

A pilot study for assessment the level of treatment adherence among hospitalized Bulgarian acromegaly patients at the University Specialized Hospital for Active Treatment in Endocrinology “Acad. Ivan Penchev,” Sofia was conducted in 2018 using patients reports. Patients' records on the regular consumption of prescribed therapy and the reasons for non-adherence were analyzed. Descriptive statistics were used to identify the number of patients adhering to the therapy, their prescribed therapy and demographic characteristics. All patients with acromegaly were asked to take part in the study. They provided signed written informed consent at their admission authorizing the use of their pseudonymized data for scientific purposes. The local hospital ethics committee approved the study (4/09.08.2019).

## Results

### Literature Review

Adherence is an objective of some researchers or non-governmental organization in Bulgaria but they are focused mainly on socially significant diseases such as diabetes, hypertension, asthma, HIV and chronic obstructive pulmonary disease (20–37). We have not identified any study aimed at analyzing the specifics of adherence among patients with rare diseases. Our previous systematic review (SR) of the scientific literature examined the level of adherence, compliance and persistence and the determinants of non-adherence in acromegaly patients in general (9). Eleven studies, which strengths and weaknesses were assessed through STROBE checklist, were included in this SR based on screening of 165 identified studies in the databases. Study sample sizes range from 1 to 1308 as the adherence rates vary between 60.7 and 92.1% for pegvisomant, 87% for lanreotide depot, and 89% for octreotide LAR. The main factors for non-adherence and loss of follow-up are side effects (100%), lack of symptoms (70.6%), financial problems (5.9%; 89%), medication discomfort (56%) and lack of motivation (23.3%). Acromegaly patients treated with long-acting SSA or pegvisomant have high level of adherence due to convenience of administration, the facilitated treatment regimen and achieving a satisfactory response. The systematic review have not identified any studies on Bulgarian acromegaly patients level of adherence and emphasizes the need for more adherence studies among heterogeneous subgroups of patients on different therapeutic regimens - mono- or combination therapy, as well as in more detail exploring the possibilities of using interventions to optimize adherence (9, 38–48).

A consensus on issues regarding therapy of acromegaly is developed in Spain in 2018. The experts agreed on that acromegaly patients should be informed about the therapy costs for the purposes of assuring treatment adherence. It is emphasized that education is one of the main factors for achieving successful treatment and provision of high level of adherence to treatment (49). Clinical guideline on “*Medicines adherence: involving patients in decisions about prescribed medicines and supporting adherence”* was developed by National Institute for Health and Care Excellence (NICE) and published in 2009. It is not focused on specific disease but gives the main principles and methods for patients involvement in the therapy and limiting the risk for non-adherence (4, 50). However, no separated guidelines for evaluation, monitoring, reporting and/or improving adherence in acromegaly patients has been found in the literature. Plunkett and Barkan developed a dialogue map involving patients, nurses, and physicians for the purposes of optimization and improvement of treatment initiation, adherence, and persistence in acromegaly patients (51). The authors highlighted the importance of education programs and communication for achieving the therapeutic goals (51).

### Bulgarian Policy and Adherence Guidelines

Adherence issues are not in the focus of most of policy or legislative documents in Bulgaria. Requirements for regular assessment of adherence level and application of appropriate methods for improvement and monitoring are not sufficiently formulated and mandatory. The issues related to treatment adherence are mentioned at different level in several documents (Table 1):

1. The Ordinance on the terms, rules and procedure for regulation and registration of prices for medicinal products from 2013 requires data for adherence improvement only for the purposes of Health Technology Assessment for any new medicinal product-candidate for reimbursement.
2. According to the National Health Insurance Fund (NHIF) requirements for treatment (initiating and continued) of acromegaly in ambulatory settings adopted in 2020, acromegaly patients should declare and sign written informed consent that they will follow the prescriber recommendations and not change arbitrarily the prescribed therapy paid by the NHIF.
3. Good Pharmacy Practice define the role of the pharmacists in the process of treatment but without giving any algorithms and without providing the best practices for assessment and improvement of adherence. In case of initiation of therapy the pharmacists should check whether the patient understands the type of prescribed therapy and how to take it. During the follow-up period the pharmacists should monitor whether the patient takes the therapy as it was prescribed by the physician. Moreover, the pharmacist ensures adequate education and information and assist for solving of various drug-related problems.
4. Good Medical Practice states the physicians' responsibilities to inform their patients about all risk and benefits associated with the prescribed therapy, to inform patients in a way they understand and make sure that the patient understands the benefits and risks of the treatment as well as to ensure effective communication with their patients for achieving efficient care and for establishing a relationship of trust.
5. According to Good Healthcare Professionals Practice, all health care specialists (nurses, midwives etc.) should participate in collection, storage and analysis of information, which is the basis for periodically evaluate the quality of health care and use the analysis to improve their practice.

**Table 1:** Analyzed policy documents.

**Document**	**Objectives**	**Adherence issues**
Good medical practice, 2020	A set of rules for diagnostic and therapeutic activities in making diagnostic and treatment decisions by physicians.	✓ The term “adherence” is not mentioned; ✓ Effective communication with patients related to prescribed therapies.
Good healthcare professionals^*^ Practice, 2020	Based on the ethical aspects and the professional behavior of healthcare professionals	✓ The term “adherence” is not mentioned; ✓ Nurses inform patients for the procedures and treatment and provide education for patients and their relatives ✓ Include patients in making decision for the provided health care activities.
Good pharmacy practice, 2020	Provision of high standards for pharmaceutical care. Contains rules for professional attitude of pharmacists to patients, self-control etc.	✓ The term “adherence” is not mentioned; ✓ Initiation of therapy - The pharmacists should check whether the patient understands what therapy is prescribed and how to take it; ✓ Continued therapy - The pharmacists should monitor whether the patient takes the therapy as it was prescribed. ✓ The pharmacist ensures adequate education and information. ✓ The pharmacist assist for solving of drug related problems.
National health strategy, 2020	Strategic document that specifies the goals for healthcare system development (till 2020)	✓ The term “adherence” is not mentioned; ✓ Centers for prevention, diagnosis, treatment, follow - up and rehabilitation of patients with specific diseases. ✓ Goal is to implement the concepts of pharmaceutical care, consulting patients for prescribed medications. ✓ Programs for rational medicines use.
National health strategy, 2021–2030 (project)	Strategic document that specifies the goals for healthcare system development (2021–2030)	✓ The term “adherence” is not mentioned; ✓ M-Health and e-Health are priorities stated in the National Drug Policy. ✓ Improving access and sharing treatment data for statistical, research or treatment purposes and encouraging patient feedback on the quality of healthcare services.
Pharmacotherapeutic guideline for treatment of endocrinology diseases, 2019	Evidence-based algorithm for treatment of endocrinology diseases (incl. acromegaly)	✓ The term “adherence” is not mentioned;
Law on the medicinal products in human medicine, 2007	Legislative framework for the pharmaceutical sector	✓ The term “adherence” is not mentioned; ✓ The pharmacy is defined as a health establishment for giving consultation.
Ordinance on the terms, rules and procedure for regulation and registration of prices for medicinal products, 2013	Rules for pricing of medicines, procedure for inclusion of medicines in the Positive Drug List, monitoring the effect of therapies, requirements for HTA	Mentioned in Annex 6 to Article 35, paragraphs 3 and 6 “Guidance on requirements for the contents of the Health Technology Assessment analysis:”“*The added value of the therapy with the new health technology, such as improved safety, **improved adherence to the therapy** compared to alternatives, as measured using specific clinical indicators against existing alternatives, must be clearly justified.”“All adverse events of clinical or economic relevance (e.g., affecting patients' quality of life, mortality, **adherence to therapy**) should be included in the analysis…”*
National health insurance fund requirements for treatment of acromegaly in ambulatory settings, 2020	Includes criteria for initiating therapy with Octreotide, monitoring and continued treatment with Octreotide, Pegvisomant or combination therapies	✓ Declaration signed by patients for following the therapy plan and not changing the therapy; ✓ No defined tools/strategies for assessing adherence.

* *Nurses, midwives, pharmacy assistants, etc*.

### Adherence Among Bulgarian Patients With Acromegaly

**Figure 1:**
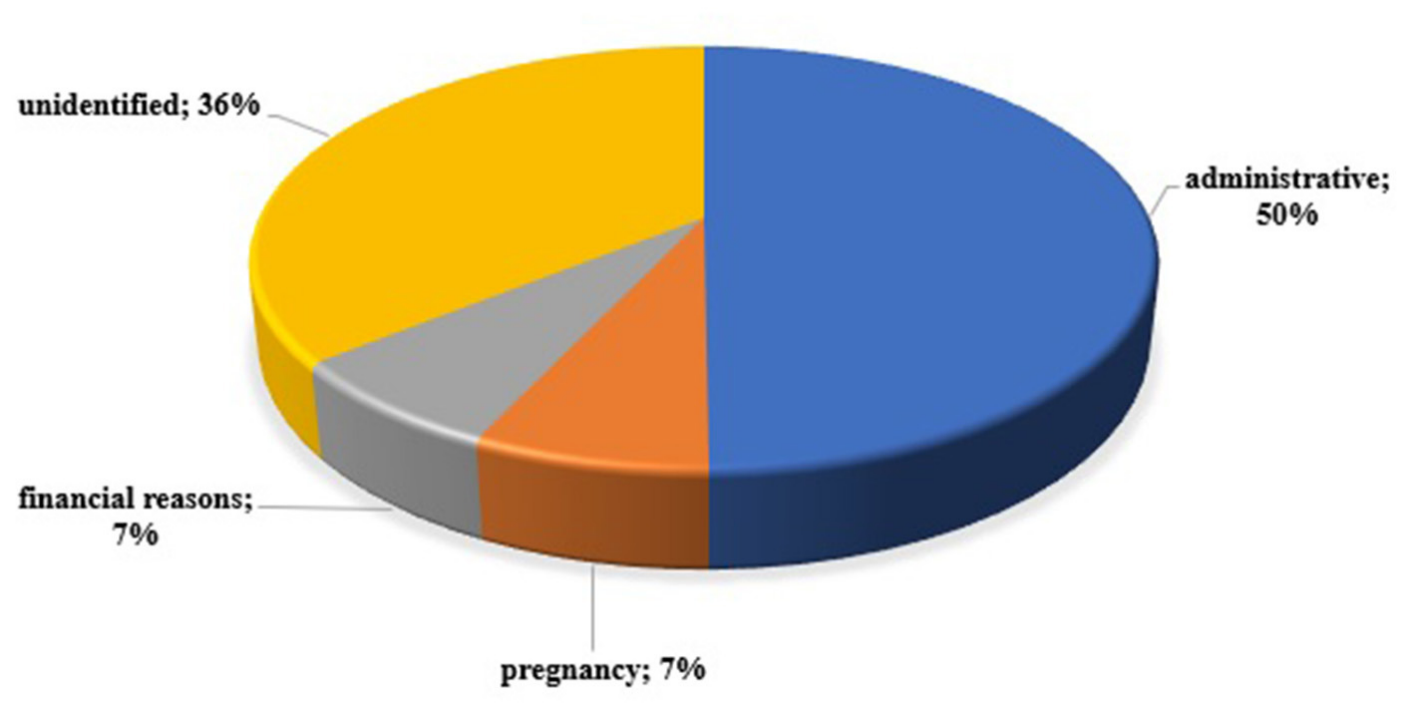
Reasons for non-adherence in the pilot study among acromegaly patients.

The study included all patients with acromegaly (*n* = 130) treated at the University Hospital “Acad. Ivan Penchev”, Sofia in 2018, and to whom pharmacotherapy was applied. Patient characteristics are presented depending on adherence to therapy. 89% (*n* = 116) adhere to and regularly take their prescribed therapy. Only 14 patients (11%) reported irregular drug therapy for various reasons, the main of which were administrative barriers (lack of therapy on the market, most likely due to export issues) (Figure 1). Financial difficulties and pregnancy have been cited as causes by two patients. For half of the patients, the cause of non-adherence was not identified. Half of those who do not adhere to drug therapy were men with a mean age of 50.43 years and 50% - women with a mean age of 52 years. The average duration of the disease in the two groups (adherent and non-adherent) was comparable: 10 and 8.5 years. The number of comorbidities and the type of therapy was also comparable between the two groups – respectively, 3 and 2 concomitant diseases; 68% of adherent patients and 79% of non-adherent were on monotherapy; 32 and 21%, respectively, were on combination therapy (Table 2).

**Table 2:** Patients' characteristics.

	**Patients (%)**	**Men**	**Average age - men (years)**	**Women**	**Average age – women (years)**	**Disease duration (years)**	**Concomitant diseases - average number**	**Patients on monotherapy**	**Patients on combination therapy**
Adherence	89%	35%	48.7	65%	55.76	10	3	68%	32%
Non-adherence	11%	50%	50.43	50%	52	8.5	2	79%	21%

Logically, statistically significant more patients who followed the prescribed therapy achieved complete remission compared to non-adherent patients: 93.18 vs. 6.82% (*p* = 0.001). Similar results were observed for remission rate and retention level, as adherence to therapy logically provided a higher response rate (*p* = 0.0005).

### Structure and Content of Bulmedacro - Bulgarian Guideline for Medication Adherence Assessment and Improvement in Acromegaly. Draft Version

The Guideline is intended for healthcare professionals (physicians endocrinologists, pharmacists, nurses) and should be created on the basis of wide consensus of all involved stakeholders - patients organizations and professional organizations representatives (physicians, pharmacists, and nurses). The final content of the guideline should be based on studies specifically designed for the Bulgarian population and addressing the beliefs, attitudes of patients and healthcare professionals about the best possible adherence assessment and improvement instruments and tools suitable for the heterogenous acromegaly patients' groups. The guideline should include more details on the nature and importance of adherence measurement and improvement and should define each specific role of the healthcare professional in the process. Collecting and reporting the level of adherence should be in compliance with good practices for documenting and publishing of results of real-life studies involving patients (Table 3).

**Table 3:** Draft version of BULMEDACRO content.

**Parts of the guideline**	**Content**
*Introduction and scope of the guideline*	Who is it for? Why do we need a guideline? What is the scope?
*Adherence* - *essence and basic principles and characteristics*	What is adherence? Which are the main components of adherence (initiation, persistence, and discontinuation)?
*Other terms* - *compliance, concordance*	Definitions and characteristics of the other terms used in the literature
*Acromegaly*	Clinical manifestation, epidemiology in Bulgaria
*Acromegaly treatment*	Main therapeutic approaches and their specifics
*Systematic review of adherence studies among acromegaly patients*	Identification and analyzing the studies published in the literature
*Methods for adherence assessment among general population*	- Indirect methods - Direct methods
*Preferred methods for adherence assessment among acromegaly population*	Which methods for assessment are the most suitable for acromegaly patients?
*Approaches for improvement patients adherence among general population*	- Education; - Dosing aids: calendar blister packages, pill boxes, Webster-packs; medication calendars, reminder charts; - Regimen simplification; - Direct communication with health providers; - Adherence reminder aids such as modern technology devices etc.
*Approaches for improvement patients adherence among acromegaly population*	Which methods for improvement are the most suitable for acromegaly patients? How to involve patients in the process?
*Algorithm for healthcare professionals (physicians, pharmacists, nurses) behavior for regularly adherence assessment and improvement in different groups of acromegaly patients*	- Newly diagnosed patients on monotherapy - Patients on combination therapy - Patients who switched their therapy
*Collecting and reporting the results*	Following the GDPR rules
*Annexes*	Schemes, tables etc. of applicable methods for assessment and improvement of adherence

## Discussion

The documents identified in the results section give very briefly some legislative basis for provision of regular assessment and improvement of the adherence level among different patients groups. This fact is in contrast with NICE practice where detailed guidelines on medicines adherence and medicines optimization have been implemented with recommendations on the best practice among all patient populations and healthcare settings (52). However, NICE's guidelines do not include specific recommendations on strategies applicable for adherence assessment and improvement for any specific diseases or conditions. Marie-Schneider highlighted the increased need of continuing development and adoption of national policies which support medication adherence considering the important role of the pharmacists in delivering a service to the patients. Moreover, in countries such as Australia, Spain, Denmark, Finland, US, Switzerland and England a number of diseases-specific or generic programs for supporting medication adherence exists. In England, USA and Switzerland, programs related to medication adherence motivation and consultation with the active role of pharmacists have been adopted (53). Some states of the USA have implemented policies and requirements for reimbursement of adherence activities performed by pharmacists (54). The need for further development of more tools and algorithms for adherence assessment and improvement with the active participation of community pharmacists has been recognized by Rickles et al. (55). In Bulgaria, no specific adherence policy documents have been developed, published or adopted in the practice which determines the importance of further discussions, conferences and expert debates. Focusing on the issues of adherence is crucial in order to avoid future complications and to achieve the desired outcomes with minimum additional public resource. According to data from a cross-sectional survey among 24,000 adults with chronic illness, more than half of them forgot to take medications and almost 40% had stop treatment within a year (56). Having in consideration these data and the fact that no detailed information for the adherence level among Bulgarian population exists, the need for adequate national disease-oriented adherence policy could be defined.

Undoubtedly, the initiation of procedure for development and implementation of BULMEDACRO - BULgarian guideline for MEdication aDherence assessment and improvement in ACROmegaly is crucial. Despite the available texts in the identified legislative documents, not clearly defined tools or instruments for regular assessment and improvement of medication adherence are described. Draft documents based on consensus among patients representatives groups (patients organizations), medical specialists (general practitioners, endocrinologists, nurses, pharmacists) and academia should be prepared and published in order to create a basis for initiating a process of medication adherence guidelines, national programs or initiatives development by the responsible legislative bodies. Further studies for the level of adherence of various patients groups, existing barriers or challenges experienced by the patients for actively involvement in the therapeutic process, the physicians' attitude to the adherence issues and the best possible approaches for adherence levels improvement among acromegaly patients in Bulgaria should be conducted. Based on the studies results, more comprehensive analysis could be done and after discussions, round tables and shared experience, a comprehensive guideline could be developed. The legislative issues for adopting the guideline are complex and related mainly with the lack of specific normative texts stating the obligation of developing and adopting of such guidelines. Moreover, broader perspective for development of national adherence policy covering the whole health system should be considered by identifying the current level of adherence among different patients groups, the barriers for optimal adherence and the approaches for improvement. Having the main direction for adherence assessment and improvement, a disease-specific approaches could be developed, discussed and implemented by experts (11).

To the best of our knowledge, no similar studies among Bulgarian patients with acromegaly have been conducted. Moreover, the Bulgarian society awareness about medication adherence issues is still very poor. Therefore, our study emphasized the importance of adherence and the need for implementation of policy and national guidelines. This pilot study showed that acromegaly patients adherence to therapy is relatively high. Almost 90% of the patients reported that they strictly follow the prescribed treatment regimen. The main barrier to adherence in the study period was the lack of medicines. Most likely, this was due to the parallel export of somatostatin analogs, which makes it difficult for patients to access, and hence the possibility of adequate adherence to therapy. Our previously conducted systematic review based on a systematic search in the Internet-based scientific databases PubMed, Google Scholar, Bioseek, aimed at assessment the level of adherence, compliance or persistence with therapy or therapeutic regimen in acromegaly patients, concluded that treatment with long - acting SSA or with pegvisomant leads to high level of adherence. The main reasons for the high level of adherence is due to the ease of administration, facilitated treatment regimen, and satisfactory response (9). A follow-up nationally-based study among acromegaly patients using more specific tools to assess the level of adherence is needed. Following-up the level of adherence should be defined as a significant part of the whole therapeutic process.

The main limitation is the pilot character of the adherence assessment study and not applying more specific instrument for adherence assessment. Moreover, some patients might be lost to follow-up and therefore the total number of patients with low or no adherence could be higher. Further detailed study on adherence among acromegaly patients using specific instruments is required. The structure of BULMEDACRO guideline gives only brief overview of the possible elements included inside the document. Wider discussions and involvement of more experts in the field would lead to improvement of the guideline structure and its future content. However, our study is the first Bulgarian one focused on the adherence issues among specific patients groups with rare condition. Therefore, the study could be used as an initial step for further investigations and improvements in the field of adherence assessment and management.

## Conclusions

Adherence to therapy is one of the most important elements of the therapeutic process ensuring desired therapeutic outcomes. Neglecting its importance for the individual patients and for the whole society, could lead to additional costs for complications, hospitalization, decreased quality of life and lack of clinical improvement despite the innovative and expensive therapies for which the society pays billion euro annually through its public funds. Patients with rare diseases should be also in the focus of specifically developed national programs, initiatives and/or guidelines for regularly assessment and improvement of adherence level. Despite the difficulties and lack of adequate legal basis, successive steps initiated by different stakeholder are required.

## Data Availability Statement

Data are available from the authors upon reasonable request.

## Ethics Statement

The studies involving human participants were reviewed and approved by Local Ethics Committee in USHATE Acad. Ivan Penchev, Medical University-Sofia, Bulgaria (approval number 4/09.08.2019). Written informed consent for study participation was obtained from the patients/participants. The patients/participants provided their written informed consent to participate in this study.

## Author Contributions

MK, YR, SV, AE, and AP carried out the research. MK and YR drafted the manuscript. MK, YR, and AP entered the patients' data in a database. MK, AE, and SV participated in the study design and reviewed the paper. All the authors have provided valuable contributions to the manuscript, read, and approved the final manuscript.

## Conflict of Interest

The authors declare that the research was conducted in the absence of any commercial or financial relationships that could be construed as a potential conflict of interest.
